# Prophylaxis of Malaria

**DOI:** 10.4084/MJHID.2012.45

**Published:** 2012-06-29

**Authors:** Eli Schwartz

**Affiliations:** The Center for Geographic Medicine and Tropical Diseases, The Chaim Sheba Medical Center, Tel Hashomer 52621, Israel

## Abstract

Malaria prevention in travelers to endemic areas remains dependent principally on chemoprophylaxis. Although malaria chemoprophylaxis refers to all malaria species, a distinction should be drawn between *falciparum* malaria prophylaxis and the prophylaxis of the relapsing malaria species (*vivax* & *ovale*). While the emergence of drug resistant strains, as well as the costs and adverse reactions to medications, complicate *falciparum* prophylaxis use, there are virtually no drugs available for *vivax* prophylaxis, beside of primaquine.

Based on traveler’s malaria data, a revised recommendation for using chemoprophylaxis in low risk areas should be considered.

## Introduction

Every year, an estimated 50 million travellers visit malaria endemic areas. Some 30,000 malaria cases are reported annually in non-endemic, industrialised countries and imported malaria remains a public health problem associated with high case fatality rates.^1^ The four European countries with the greatest number of reported cases of imported malaria are the United Kingdom, France, Italy and Germany. *P. falciparum* accounts for almost 70% of all the imports.^2^

Theoretically malaria prevention could be based on **vaccine**, on **personal protection** and on **chemoprophylaxis**. However, malaria vaccine is not on the near horizon, especially not for travelers, despite some encouraging new data.^3^ Personal protection, albeit an important tool, is often not sufficient, thus chemoprophylaxis remains the principal means to prevent malaria.

## Personal Protection

Personal protection refers to all measures that can be taken to reduce the risk of the anophline bites. Since the anophleles mosquito is a night feeder, protection is relatively easy when compared for example to protection from dengue mosquitoes, which are day feeders. Protective strategies include wearing clothing after sunset that covers as much bare skin as possible, and using mosquito repellents on exposed skin containing about 35% *N, N*-diethyl-3-methylbenzamide (DEET) formulations. The use of insecticide-impregnated clothing can also be helpful.^4^ While indoors, staying and sleeping in air-conditioned rooms, and sleeping under mosquito nets provide good protection. For expatriates who live in endemic areas, eradicating mosquito breeding sites around the house is important. Strict adherence to these measures reduces the chances of acquiring malaria, but they cannot be relied upon to prevent malaria in environments where anopheline mosquitoes and infected humans are present in abundance.

Malaria in many areas of the world is seasonal and usually reaches its peak at the end of the rainy season, thus avoiding travel during peak malaria seasons may reduce the risk.

## Chemoprophylaxis

Malaria chemoprophylaxis in travelers to endemic areas is one of the most complicated and challenging aspects of travel medicine and poses several significant problems.

- Risk-benefit: the risk of malaria infection and severe consequences of the disease should be weighed against the risk to the traveler of the drug itself. Several drugs have had fatal outcomes to consumers; others have caused significant adverse events and an interruption of travel due to the adverse events. This side of the equation is not always weighed appropriately by those who prescribe these drugs.
- Cost-benefit: with the development of malaria-resistant species, new drugs have been made available, usually at a higher cost. Thus for budget travelers, especially with long-term trips, use of these drugs becomes a burden. When the malaria risk is minimal, the benefit of such an expense is often felt unjustified.
- Inadequacy of the current chemoprophylaxis: Although we use the term, “malaria prophylaxis,” in reality we have “*falciparum* prophylaxis” and not a pan –malaria prophylaxis. Travelers who take the recommended prophylactic drugs may still present with late-onset *vivax* infection.^5^ Although *vivax* malaria in most cases does not have a severe outcome, it remains a significant disease, and one that the traveler would like to prevent. Additionally, if a traveler contracts malaria despite taking prophylaxis, he or she may deem it useless and skip taking it for subsequent trips.

Adverse events, cost-benefit calculations and the inadequacy of preventing late-onset *vivax* malaria are all probable reasons for low adherence to prophylaxis, and well-known to those practicing travel medicine. A survey done in our institute of travelers presenting post-travel, seeking medical advice for any reason [n= 1207], demonstrated that only 15% adhered to malaria prophylaxis [E. Schwartz unpublished data].

These results highlight the discrepancy between the medical recommendations for malaria prophylaxis and the travelers’ perceptions.

## Principal of Chemoprophylaxis: Blood Stage vs. Liver Stage Prophylaxis

**Figure 1:**
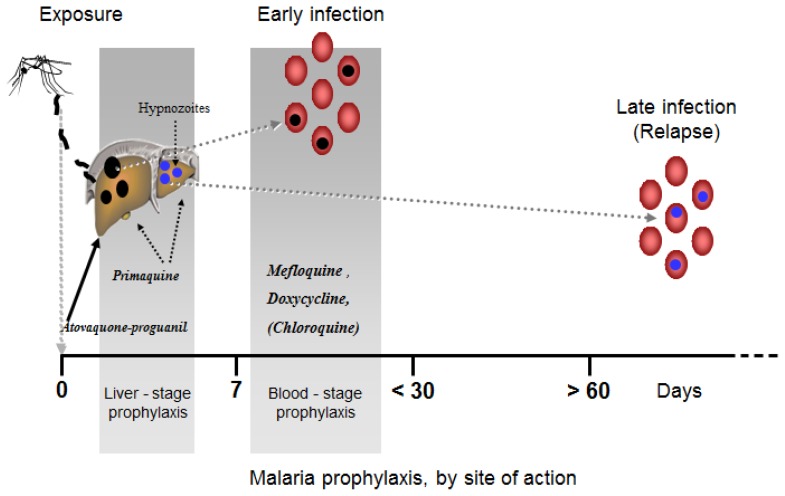
Malaria Life cycle (partial illustration). *Blood stage prophylaxis:* Drugs which act on the malaria parasites only within the erythrocytes. (such as: Mefloquine, Chloroquine, Amodiaquine). They have to be continued therefore for 1 month after leaving the malarious area. As can be seen in the figure, late infections will not be prevented. *Liver stage prophylaxis:* Drugs which act on the malaria parasites within the hepatocytes (such as Primaquine and Malarone). It is sufficient to continue the drug for a few days after leaving the endemic area. However, only primaquine potentially may prevent all types of malaria including the late infection.

The parasite’s life cycle in humans occurs in two stages (Figure 1). In the initial liver stage, or exo-erythrocytic stage, parasites multiply in the hepatocytes and eventually cause them to rupture. Two species, P. *vivax* and P. *ovale*, have persistent liver stages resulting in relapse months to years later.

The second, or erythrocytic stage occurs when the parasites are released into the bloodstream, invade erythrocytes, and cause clinical illness. This stage occur usually after 12 ± 3 days in P. *falciparum* infection and after 14± 3 days in P. *vivax* infection.

It should be noted that:

▪ The malaria parasite is different in its sensitivity to drugs in each form of its cycle. Thus a drug which acts on the parasite during the intra-erythrocytic stage will not necessarily act against it in its liver stage and vice versa.
▪ Chemoprophylaxis does not prevent the infection (as in the case of vaccine preventable diseases), but rather works as having a killing effect against the parasite, either within the erythrocytes or within the hepatocytes, thus preventing the clinical disease.

Based on the parasite’s life cycle, there are two types of malaria chemoprophylaxis, based on the site of action.

## Blood Stage (Suppressive) and Liver Stage Prophylaxis (Causal)(Figure 1)

*Blood stage* prophylaxis refers to drugs that act only on parasites within the red blood cells. These are the commonly known antimalarial drugs that have been used over the past 60 years or so. Among their disadvantages is that they must be continued for 4 weeks after travel to eliminate the parasites within the RBCs which may emerge from the liver as late as 2–4 weeks after exposure. Another major disadvantage is that since these drugs have no activity against the liver stage and development of hypnozoites, they actually prevent only primary *vivax* (and *ovale*) infection, and they do not have the ability to prevent relapse. They are therefore a complete prevention only in the case of P. *falciparum* infection.

*Liver stage* prophylaxis, refers to drugs that act on the parasite while invading the hepatocytes. Since these drugs kill the parasite early on during the infectious process, there is no need to continue taking the drug after leaving the endemic areas. For *falciparum* infection, it has the advantage of shortening the duration of the prophylaxis usage and instead of continuing medication for 1 month post-travel there is only a need to continue for several days, which may increase compliance with the full prophylaxis schedule. In the case of *vivax* (and *ovale*) infection, liver stage prophylaxis is imperative. Only drugs that act early on the liver stage and prevent the hyponozoite formation offer complete prevention of this infection. There are currently only 2 drugs which act on the liver stage: atovaquone-proguanil and primaquine, but only primaquine has cidal activity against the hyponozoites ( as discussed further in the vivax prophylaxis section).

## Falciparum prophylaxis

The introduction of chloroquine in the 1950’s brought great hope that falciparum prevention could be easily achieved with a long acting drug, that was well-tolerated and taken on a weekly basis. However, within one decade drug resistance appeared, first in South East Asia and within a few years this resistance spread throughout the endemic areas. Currently, the resistance of P. *falciparum* to chloroquine is almost universal. It remains effective only in Central America, the Caribbean (mainly Haiti), and in some of the Middle Eastern countries where the prevalence of P*. falciparum* is in any case very low.^6^

The history of the development of chemoprophylaxis since then includes trying to find new drugs that are both efficacious and well-tolerated. It should be remembered that a drug with even an infrequent severe adverse event, if used as prophylaxis for a very large volume of travelers, might quickly present as a harmful drug. Two drugs which were introduced after chloroquine, namely Amodiaquine and Sulfadoxin-Pyrimethamin (Fansidar), were excluded from use as prophylaxis due to severe adverse events, including fatal cases. With amodiaquine, fatalities were due to agranulocytosis, and with Fansidar they were due to fatal toxic epidermal necrolysis. Risk-benefit calculations that were done at that time showed that in some geographical areas, the risk of fatal outcomes from these drugs was higher than from the disease.^7^

The principal drugs currently in use are mefloquine, doxycycline, atovaquone-proguanil (Malarone), and to some extent Primaquine (Table 1)

## Mefloquine

Mefloquine (Lariam, Mephaquin) was developed from a quinolone–methanol compound at the Walter Reed Institute. It was found to have potent anti-malaria activity, including against chloroquine-resistant P. *falciparum* strains and due to its long half-life, it can be taken on a weekly basis. These characteristics of the drug created optimism, in the mid-1980’s when first introduced in Europe (and in the US in 1990), that an ideal replacement for chloroquine had been found. In addition, long-term prophylaxis usage among Peace Corps volunteers in Sub-Saharan Africa demonstrated its safety and good tolerability.^8^

However during the subsequent decades of its use, there arose several concerns.

- *Mefloquine resistance*: Resistance was occasionally reported first from the Thai-Cambodian border, followed by reports from other parts of Asia and to lesser extent from Africa and the Amazon region. The level of mefloquine resistance in the area of Thai-Cambodian and Thai-Burmese borders has reached 50%, thus precluding it from use as prophylaxis in this specific region. While in all other regions, the resistance level currently is more anecdotal and the drug can be used in these areas.
  However, the main concern for travelers regarding the use of mefloquine is its safety and tolerability.
- *Mefloquine safety and tolerability:* Mefloquine’s adverse side effects may include neuropsychiatric, gastrointestinal, and less commonly dermatological events.

The neuropsychiatric adverse events (AE) associated with mefloquine are the worrisome complaints, and have received a vast amount of public attention, (probably more than any other malaria prophylactic drug).

The neurological disorders include headache, dizziness, confusion, vertigo and seizures. Peripheral neuropathies such as paresthesia, tremors and ataxia have also been reported.

The psychiatric disorders may include insomnia, strange dreams, restlessness, anxiety, depression, and psychosis.

The incidence of any AE due to the drug is hard to assess since results varied and ranged from about 10%–90% depending on the study design and whether a comparator was used.^9^ The rate of drug withdrawal also varied, from 0.9% to 5%.^10^,^11^

The most concerning issue of chemoprophylaxis is the rate of serious AEs, resulting in a possible life threatening condition, or causing severe disability or prolonged hospitalization. Well-designed prospective studies of mefloquine’s adverse events may not identify a significantly higher number of events in comparison to other anti-malaria drugs, because of the small number of participants, and also it is easy to miss the relatively rare severe adverse events.^10^,^11^,^12^ Only post-marketing surveillance studies (with their limitations) have sample sizes large enough to capture the rare serious adverse events, thereby drawing significant conclusions.

Results of a study done by questionnaire among mefloquine users in British soldiers showed a rate of severe AEs as 1:6000,^13^ while a questionnaire among European travelers showed a rate of 1:10,000.^13^ Spontaneous reporting among Canadian travelers demonstrated a rate reaching 1:20,000.^9^

Mefloquine AEs as reported in all studies are more common in women. In most cases, susceptible individuals have problems after the first 1–3 doses.^15^ The recommendation therefore is to start mefloquine about 2 weeks prior to departure in order to assess any adverse effects which may necessitate the use of an alternative prophylaxis.

In a case control study among travelers with serious AEs due to mefloquine prophylaxis, no difference in the level of mefloquine in the blood was found between the patients and the control groups. Also, no significant difference was found between mefloquine levels in the blood of men and women. These results suggest that blood levels of mefloquine do not correlate with its severe adverse events.^15^

### Pregnancy

One of the advantages of mefloquine is the fact that this is the only drug that can be taken during pregnancy when traveling to chloroquine-resistant areas. It is officially recommended for the 2nd and 3rd trimesters of pregnancy. Limited data also suggest that its use during the first trimester is safe. Therefore, mefloquine should be recommended to a pregnant woman who cannot avoid traveling to endemic areas during her first trimester.^16^

### Contraindications

Due to the possible drug-associated neuropsychiatric effects, it is contraindicated in travelers who have seizure disorders. In addition, it should not be given to travelers with active psychiatric disorders such as depression, anxiety, psychosis or any other major psychiatric disorders. It is advisable not to prescribe this drug to patients with a history of the above-mentioned psychiatric disorders, even if they are currently stable.^16^

Since the drug is related to quinine, it should not be given to persons with a known hypersensitivity to mefloquine or to quinine compounds. It is also not recommended for travelers with cardiac conductions abnormalities.

## Doxycycline

Doxycycline, a synthetically derived tetracycline, is a highly effective drug for the prevention of malaria. In studies conducted in non-immune populations, the dosage used was 100mg daily, resulting in greater than 95% efficacy against P.*falciparum*, indicating that it is as efficacious as the other drugs currently available, such as mefloquine and atovaquone–proguanil.^17^ Although it has some liver-stage activity, its main action is on the erythrocytic stage thus requiring 4 weeks of continuation of the drug after leaving an endemic area.

Malaria resistance to doxycycline has not been reported yet in any of the malaria endemic areas.

### Tolerability

The most common adverse events are gastrointestinal-related complaints such as abdominal pain, nausea, vomiting and diarrhea. A severe complication is esophageal ulceration, and therefore the recommendation is to take it in an upright position, with food or full glass of water and not before bedtime.

Dermatological complications include photosensitivity, which is a concern for the travelers exposed to the sun in tropical countries. The reported dermatological complications vary and may reach 21%,^17^ although in a four-arm multicenter randomized controlled trial comparing doxycycline, mefloquine, atovaquone-proguanil and chloroquine –proguanil, in travelers to Africa, skin reactions with doxycycline were less common than with chloroquine–proguanil.^12^

An important adverse effect of the drug among female travelers is the risk for vaginal candidiasis, which estimated to occur in 2.8%.^18^

The requirement to take doxycycline daily and the fact that it must be continued for one month after leaving a malaria endemic area, are also drawbacks in terms of its use.

An extra-benefit of using doxycycline for malaria prophylaxis might be its preventive measure against leptospirosis, which is a common hazard in the tropics, and its potential protection against rickettsial diseases and traveler’s diarrhea.

*Contraindications* are for pregnant women, breastfeeding mothers, children under 8 years old, and those with a history of allergy to any of the tetracycline classes.

## Atovaquone-Proguanil

The spread of drug –resistant *falciparum* malaria, and the widespread reluctance to use the known anti-malaria drugs due to their side effects, led to the pursuit of new antimalaria drugs. Atovaquone-proguanil (Malarone) is the latest anti-malarial drug to be developed.

This drug is well-tolerated, and has good efficacy for resistant *falciparum* strains.

An added advantage of this drug is the fact that it acts on the *liver stage* of the malaria parasite thus shortening considerably the amount of time needed to continue it post-travel (Figure 1). It is therefore the first liver-stage drug since the introduction of malaria chemoprophylaxis, (with the exception of Primaquine, which will be discussed below).

The drug is a fixed combination of Atovaquone 250 mg and of Proguanil 100mg. Pediatric tablet contain the same combination with a quarter of the dose of each component (62.5/25 mg).

Atovaquone alone was well-established drug against *Pneumocystitis carinii*.

Its mode of action against the plasmodia spp. is via inhibition of the mitochondrial electron transport system, at the level of cytochrome-b complex.

Proguanil is an old anti-malaria drug, which acts by inhibiting the parasite’s dehydrofolate reductase.

Each of these drugs has weak anti-malarial activity but in combination there is a synergistic effect, with an efficacy of 95–100%.^19^ Each of these drugs’ components was tested separately in human volunteers and found to be active at the liver stage.^20^,^21^ The fixed combination atovaquone-proguanil was also tested in human volunteer challenge trials where non-immune subjects were given 1 tablet of this combination for 8 days, starting 1 day before the mosquito challenge and continuing for 7 days after. None of the subjects (n=12) who took the active drug developed malaria, while all (n=4) who took placebo developed *falciparum* malaria.^22^ This clearly demonstrated that the combination of the drugs has good activity against the liver stage of P. *falciparum*. This study was the basis for recommending the drug atovaquone-proguanil to be continued for 7 days after leaving the endemic areas. However, a very recent study done as the same method described above showed that even taking it at the last day should be enough.^23^

## Tolerability

Several studies of atovaquone-proguanil have been conducted among travelers to evaluate its safety and tolerability in comparison to other antimalrial drugs. In a four-armed multicenter randomized controlled trial comparing the 4 drugs commonly used in travelers, namely mefloquine, doxycycline, chloroquine-proguanil and atovaquone –proguanil, the latter had the lowest withdrawal rate due to adverse events (2%).^12^ Other studies where only one drug was used as a comparator, either mefloquine or chloroquine-proguanil, the atovaquone-proguanil had a better safety profile.^11^,^24^

The drug has been in use for about a decade and seems to maintain a very good safety and tolerability record. However, the main drawback for using it is the higher cost in comparison to the other anti malarial drugs, which obviously increases with increase the length of travel.

## Adverse events

The most common adverse events are related to gastrointestinal complaints, such as abdominal pain, nausea or vomiting and therefore it is recommended that it be taken with a meal.

Dermatological complaints such as rashes and pruritus may occur, probably due to the proguanil component.

## Indications

Atovaquone-proguanil is indicated for P. *falciparum* prohylaxis. In the US, it is indicated without a time limitation, meaning that long–term travelers, expatriates and military personnel on long-term missions can use it. In several countries in Europe, its use is limited only to short-term travelers (30–90 days), since data on its safety with prolonged use are lacking.

It is indicated for children above 5 kg, but dose should be modified according to weight (Table 2).

## Contraindication

The drug is contraindicated in patients with severe renal failure (creatinine clearance <30 mL/min), and in those with known allergies to one of the drug components.

The drug is contraindicated in pregnancy, since there is not sufficient information about it safety in pregnancy.

## Primaquine

Primaquine, as viewed by many clinicians, has its only role in regard to its activity against P. *vivax* infection. However, since its introduction in the early 1950’s, primaquine has been found to be active against the early liver stages of both P. *falciparum* and P. *vivax* malaria.

Primaquine is an 8-aminoquinoline and was developed in the 1940’s. In a study conducted in 1954, healthy volunteers who were inoculated with P. *falciparum* malaria but were given primaquine, at a daily dose of 30 mg before the sporozoite inoculation, the infection was prevented.^25^

Despite the fact that primaquine was highly effective against the early liver stages of the parasite (P. *falciparum* and P. *vivax*), it never gained widespread use as chemoprophylaxis. This was most likely for two principal reasons. The first was the reporting of severe adverse effects, including methemoglobinemia and hemolytic anemia occurring in glucose-6-phosphate dehydrogenase (G6PD)-deficient patients.^26^,^27^ The second reason was perhaps due to the introduction of a new drug, chloroquine, which was relatively safe and highly potent.

In recent years however, primaquine has made its comeback as prophylaxis and not just for the radical cure of *vivax* malaria.

The first study of primaquine used as prophylaxis was conducted in Kenya among a local population, in a hyperendemic area, known to have a 90% incidence of new cases of *falciparum* malaria and with an estimate of nearly one infective mosquito bite per person per night. The efficacy at the end of a 3-week follow-up period was 85% for primaquine, 84% for doxycycline, 77% for mefloquine, and 54% for chloroquine/proguanil.^28^

Another study was conducted in Irian Jaya (northeast Indonesia), an area endemic for both P. *falciparum* and P. *vivax* malaria, with a population of transmigrants who were most likely non immune. After 52 weeks, efficacy against P. *falciparum* relative to placebo was 94.5% for primaquine and 33.0% for chloroquine, and efficacy against P. *vivax* was 90.4% for primaquine and 16.5% for chloroquine.^29^

A similar study was conducted in 1997 with Colombian soldiers.^30^ In the primaquine group, the protective efficacy was 94% against P. *falciparum*, and 85% against P. *vivax*. Another study, again with transmigrants to Irian Jaya, showed similar results. Participants received 20 weeks of primaquine or placebo. Primaquine showed an overall protective efficacy of 93%, with> 92% protective efficacy against P. *vivax* and 88% against P. *falciparum*.^31^

## Tolerability

The most common adverse effects of primaquine are gastrointestinal effects that are dose dependent. In studies done during the early 1950’s, it was found that doses of up to 30 mg/day were associated with minimal gastrointestinal upset and only doses of 45 mg/day or higher were associated with a significant rate of adverse effects.^7^

Recent studies also have shown minimal adverse effects. In the Colombian study,^25^ two subjects (2%) who were taking the drug withdrew from the study because of gastrointestinal complaints. In the Indonesian study,^27^ primaquine was taken daily for about 1 year, with no withdrawals from significant adverse events. Complaints were similar in the placebo and drug groups.

In the author’s study among travelers,^32^ primaquine was well tolerated. There was only one case of withdrawal, which was due to nausea and vomiting (a rate of 1 per approximately 200 cases).

Primaquine has gained more recognition in recent years and was listed in Canada and the US as an option for malaria prophylaxis. Its role was reemphasized in a report from a CDC expert meeting on malaria chemoprophylaxis^33^

## Toxicity

Primaquine can produce marked hemolysis when the drug is administered daily to individuals with G6PD deficiency; therefore, testing for G6PD before treatment is necessary.

Methemoglobinemia occurs in normal individuals, but without clinical significance.

## Dosage and Recommendation (Table 1)

Since primaquine is a drug that acts on the liver stage of the malaria parasite, there is no need to continue taking it for 1 month after departure from the malarious area (opposite to most of the other antimalarial drugs which mentioned above, that act on the erythrocyte stage of the malaria parasite). Therefore, the traveler should start taking it 1 day prior to entering the malarious area and to continue taking it daily for 3–7 days after departure from the malrious area. The recommended dose is 30mg (2 tablets) per day for adults. Due to the short half-life of primaquine, it must be taken daily, preferably with food to avoid gastrointestinal upset.

The CDC recommends taking it for 7 days after departure from the malarious area.^33^ Other authorities recommend it for only 3 days after cessation of exposure.^34^

The pediatric dose is 0.5 mg/kg/day.

*Pregnant women* should not take it due mainly to the fear of G6PD deficiency in the fetus. Lactating women can use it if the infant has been tested for G6PD.

## Special Populations

Special populations who may need particular attention are pregnant women, and children for whom not all drugs mentioned above can be recommended. (Table 2). Breast feeding mothers should know that the amount of anti-malaria drugs excreted in the milk is not sufficient to offer protection to the child, on the other hand it will not likely be harmful even with drugs that are not approved for small children.^35^

Another special population are long-term travelers (usually considered those travelling >6 mo.), or expatriates who remain for several years in endemic areas. Two questions arise; which drug is considered to be safe for long-term use, and secondly, what is the best approach either taking chemoprophylaxis continuously, or use only personal protection measures and seek medical care in the case of a febrile illness.^36^

Mefloquine, and chloroquine (which is of no value in most areas of the world ) are the only drugs that have a good long-term follow-up record^8^ (Table 2). Atovaquone-proguanil, although recommended by the CDC for long-term use, has never been assessed in large numbers, and maximal period of observation was 34 weeks.^37^

The safety of doxycycline has been demonstrated in patients taking it for long periods for acne, for malaria prophylaxis there are reports of long-term use among soldiers taking it for up to 1 year.^38^

The safety of long term primaquine use was tested in Indonesia where it was given for 52 weeks.^29^

It is evident that expatriates who live for long periods of time even in Sub-Saharan Africa typically do not take malaria chemoprophylaxis continuously, but rather rely on identifying symptoms and seeking medical care (usually available and known to them) when needed. However, even in these cases it is advisable to use chemoprophylaxis, at least at their first few months of their stay.

Long term travelers who are traveling in endemic areas and moving from one place to the other including remote areas, should be encouraged to take chemoprophylaxis continuously throughout their trip especially when it is done in Sub Saharan Africa.

## Vivax prophylaxis

*P. Vivax ( it relates to P. ovale as well)* has a more complicated life cycle than *P. falciparum* due to the formation of liver hypnozoites, which can result in a clinical relapse several months after the primary infection. Therefore, complete prevention of this infection is much more challenging and can be achieved only if both primary and late infections are prevented.

The life cycle of *P.vivax* has a bimodal incubation time:

- The primary attack, which follows exposure to infectious sporozoites, occurs about 14± 3 days after the mosquito bite ( for *P. falciparum* this incubation time is of 12± 3 days).
- The late infection is a relapse following activation and maturation of the dormant liver stage hypnozoite (Figure 1).

The chance of, and incubation time for relapse largely depends upon the geographic origin of the infection. The tropical *P. vivax* strains tend to have a higher probability of relapse (>30%), a shorter period between primary attack and relapse (17–45d), and a higher incidence of multiple relapses (>2), while the temperate strains (such as the Korean strain) tend to have fewer relapses, and a longer period before relapse, about 9 months.^39^ Thus, clinicians should be alert to the possibility of *vivax* malaria attacks several months or even a year or more following travel to an endemic area.^5^

## Blood Stage Prophylaxis In Vivax Malaria

Blood stage prophylaxis is the most common type of prophylaxis in use. Chloroquine, was the first drug in this group to be extensively used. It was introduced in the early 1950’s for the prevention of both *falciparum* and *vivax* malaria. While chloroquine-resistant *P. falciparum* appeared quite quickly, in the late 1950’s, chloroquine-resistant *P. vivax* presented only in the late 1980’s. It is a significant problem in eastern Indonesia where more than half of infections with *P. vivax* appears to be resistant. Resistance has been occasionally reported from other areas in Southeast Asia, South Asia, and in South America.^40^

Mefloquine and doxycycline, are also common blood stage drugs for prophylaxis and are effective against p. *falciparum* and found to be effective against *vivax* malaria as well. During the 1990’s, well-controlled trials of all of these drugs were conducted in northeastern Indonesian New Guinea, where *vivax* malaria is heavily endemic and notoriously resistant to chloroquine. They demonstrated 100% protective efficacy.^41^ Since these drugs have no activity against liver stages and development of hypnozoites, they actually prevent only *primary infection* and not *late relapses*. In fact in recent years, with the increase of travel to the tropics, it has become more evident that using recommended prophylaxis, which is almost exclusively blood stage prophylaxis, only postpones the first clinical attack of malaria to several months after return. This was clearly demonstrated in a study among US and Israeli travelers where the majority of all imported *vivax* cases [60–80%] were late infections (more than 2 months after return) in travelers who took recommended prophylaxis. This clearly illustrates the deficiency of the currently recommended prophylaxis in fully preventing *vivax* infection.^5^

The common recommendation of chloroquine use for *vivax* prevention is based on the sensitivity of *vivax* spp. to chloroquine, but it ignores the fact that chloroquine can not prevent the hypnozoite formation and therefore can not prevent late infection.

Thus, the role of chloroquine or other blood stage prophylaxis in complete prevention of *vivax* is very limited (it might have some value only in areas where the relapse rate is very low), and ***should not*** be regard as *vivax* prophylaxis.

**Figure 2:**
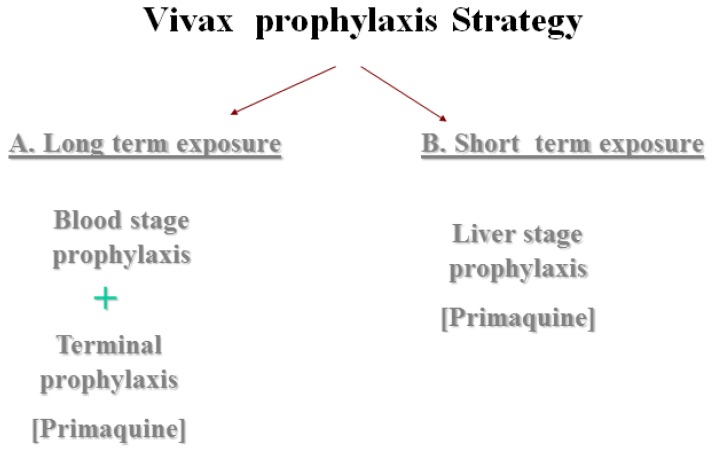
*Vivax* prophylaxis Strategy

To overcome this problem there are 2 options (Figure 2); one is by adding ***Terminal prophylaxis***, meaning presumptive standard treatment with primaquine upon leaving an endemic area. The term “presumptive anti-relapse therapy” (PART) has been proposed to better describe this treatment strategy.^33^ It is intended to kill latent liver stages of *P. vivax* and thus prevent relapse. The dose of primaquine for this purpose is under re-evaluation. The common knowledge of dosing with 15mg daily for 14 days, is probably insufficient, especially for the high body weight of typical travelers from industrialized countries. The current preferred recommendation is a single 30mg dose of primaquine (base) taken daily for 2 weeks after leaving the endemic area.

There remains with this approach a grey area regarding which travelers would benefit. Should everyone who was in a malaria endemic area where there is p. *vivax* take it, or should it be reserved for high risk populations, such as long term travelers or those who have been to highly endemic *vivax* area (such as in our experience the Omo region in Ethiopia).

The second approach and more convenient one is by using ***Liver stage Prophylaxis***. This prophylaxis can eliminate both primary attacks and relapses of P. *vivax* and can be effective for P. *falciparum* prevention as well. Primaquine is the only available drug known to have this prophylactic activity against *vivax* malaria. Atovaquone-proguanil, despite being a known liver stage prophylaxis against *falciparum* malaria (as mentioned above), does not prevent late *vivax* infection. Although studies, which looked at the efficacy of the drug for *vivax* malaria, found it to be 82% efficacious in Indonesia and 100% in Colombia.^42^,^43^ However both studies evaluated its efficacy only for primary infection (a follow- up of 1 month after exposure). Recent evidence from Israeli travelers to Ethiopia (Omo region) has shown the inefficacy of this drug to prevent late infection. Although during the first month post travel the efficacy of the atovaquone-proguanil was 100%, the relapse rate among the users was 56% during 1 year of follow up, similar to blood stage drugs [E. Schwartz, submitted for publication].

On the other hand, primaquine studies of the last 10 years show effective protection against primary attacks in transmigrants in Indonesia and in travelers.^44^ In travelers, long-term follow up demonstrated its efficacy also in preventing relapse.^32^ Our above mentioned study showed that in the highly endemic area of Ethiopia, while the malaria attack rate among non-primaquine users (mefloquine, doxycycline and atovaquone-proguanil) was about 50%, in primaquine users it was 1.4% [E. Schwartz, submitted for publication ].

Since the early clinical trials of primaquine demonstrated its activity against *falciparum* malaria as well,^25^ it can be used as a single agent for all malaria species.

The dose and contraindications are mentioned above (Table 1).

In conclusion, for travelers to *vivax*-predominant areas; short-term travelers, a daily dose of primaquine (only if G6PD is normal) seems to be the most convenient option.

For long-term travelers, a weekly dose of chloroquine (depends on the area), or of mefloquine (if there are no contraindications) followed by terminal prophylaxis with primaquine, would be the most convenient and efficacious (Figure 2).

## The Threshold for Malaria Chemoprophylaxis Use

Malaria chemoprophylaxis should not be used in areas where there is no malaria., therefore, the practitioner who sees the traveler prior to his departure should be familiar with the non-endemic areas. It should be remembered that even within endemic countries, there are often areas free of malaria. For example, travelers who trek in Nepal are not at risk due to the high altitude. The same holds true for travelers to high altitude areas even within Sub-Saharan Africa such as Addis Ababa and the Ethiopian plateau which are highland areas above 2000 m., etc.

In the endemic areas, the risk for travelers varies significantly. The higher risk for *falciparum* malaria is in West Africa, estimated to be 2.4% per month of stay, while in East Africa is 1.5% per month of stay, risk is also high in travelers to the Pacific islands (Solomons and Papua New Guinea), but is 10–20 times less in travelers to Asia, and 30–40 times less in travelers to Latin America.^45^

Thus, the more complicated issue is the decision about malaria chemoprophylaxis in those who travel to low risk areas, and what should be decided as the threshold(if any) for using chemoprophylaxis. There is no consensus yet about this issue. According to the policy of the US CDC, the world is divided to “All or None” in regard to recommending chemoprophylaxis; one should either take it or not.^35^ The WHO introduced another category for certain areas of the world and the recommended policy is personal protection only [named: type 1 prophylaxis].^16^

The real challenge in travel medicine is making recommendations for low risk malaria areas such as Central and South America, and several parts of East Asia. In these regions the risk of malaria definitely exists and there are cases of imported malaria from those areas, however the overall risk for travelers is low. A study from Europe suggests that the risk of adverse events from hemoprophylaxis is likely to be significantly higher than the risk of acquiring malaria in the most popular tourist destinations in Central and South America.^6^

A similar conclusion came from an analysis of malaria imported into eight European countries from the Indian sub-continent (ISC) (India, Pakistan, Bangladesh and Sri Lanka).^46^ The proportion of cases from the ISC ranged from 1.4%–4.6% of total imported cases, and again *P. falciparum* cases accounted for only 13% of all cases from the region. Thus, the calculated risk of malaria in UK residents visiting the region was > 1 case per 1,000 years exposed.^46^

Therefore, the TropNet group recommends that the non-selective prescribing chemoprophylaxis for visitors to the India subcontinent, should be dropped.^46^

An alternate strategy adopted by a number of European countries, for example Switzerland,^47^ is to provide travelers with **“emergency standby treatment”** to be used in case malaria symptoms occur during travel. This assures treatment of a life threatening attack of *falciparum* malaria, and avoids adverse events associated with excessive chemoprophylaxis

There is no consensus about the use of stand by therapy. However the strategy of bite prevention measures remains important, as these are effective, safe and have the added benefit of reducing other vector borne diseases. In addition, travelers have to be educated to seek medical advice in the case of a febrile illness.

## Figures and Tables

**Table 1:** Use of anti malarial drugs for P. *falciparum* prophylaxis.

Drug	Dose (adult)	Dose regimen	Beginning of prophylaxis (before exposure)	End of prophylaxis (after exposure)
Atovaquone-proguanil	250 mg/100mg	Daily	1 day	7 days
Mefloquine	250 mg	Once a week	1–3 weeks	4 weeks
Doxycycline	100 mg	Daily	1–2 days	4 weeks
Primaquine * *G6PD testing is mandatory before its use*	30 mg (base) [usually =2 tabs]	Daily	1 day	3–7 days
Chloroquine	300 mg (base) =500 mg salt	Once a week	1 week	4 weeks

**Table 2:** Features of the main drugs used for *P. falciparum* prophylaxis

Drug’s name	Site of action	P.f efficacy	Adverse Event profile	Use in Pregnancy	Pediatric use	Reported Long term use
Chloroquine	Blood stage	Usually not [only central America & Haiti]	Low	Yes	Yes, all ages	30 mo.
Mefloquine	Blood stage	+ [resistance in S.E Asia]	High [mainly neuro-psychiatric]	Yes from 2nd trimster. Limited data on 1st trimster	Yes, above 5 kg	30 mo.
Doxycycline	Blood stage	+	Low, GI, Vaginal discharge	No, teratogenic	Yes, only >8 years old	12 mo.
Atovaquone-proguanil (Malarone)	Liver stage	+	Low, GI	No, No enough data	Yes, above 5 kg	8 mo.
Primaquine	Liver stage	+	Low, G6PD needed	No, G6PD status of fetus is unknown	Yes, all ages	12 mo.
